# Efficacy of Trastuzumab + Cisplatin Combined with Irinotecan on the Quality of Life of Patients with Advanced Her-2 Positive Gastric Cancer

**DOI:** 10.1155/2022/8762647

**Published:** 2022-08-27

**Authors:** Yu-Xin Yang, Chun-Ying Li, Wen-Jie Yin, Xia Chen

**Affiliations:** Gastroenterology Ward III, Cangzhou Central Hospital, Cangzhou, Hebei Province, China

## Abstract

**Objective:**

To observe the effect of trastuzumab and cisplatin combined with irinotecan in the treatment of advanced Her-2 positive gastric cancer and its influence on disease control rate.

**Methods:**

From January 2018 to January 2021, 120 patients with advanced Her-2 positive gastric cancer admitted to our hospital were selected as the research subjects. According to the treatment plan of the patients, they were divided into a control group and a joint group, with 60 cases in each group; the control group was given trastuzumab + cisplatin, the joint group was treated with irinotecan on this basis, and the clinical effects and disease control rate of the two groups were observed.

**Results:**

After treatment, there were 4 patients with CR in the joint group and 0 patients with CR in the control group. The ORR and DCR of the joint group were significantly higher than those of the control group (*P* < 0.05). The expression levels of CA199, CEA, and CA724 after treatment in the two groups were significantly reduced (*P* < 0.05), and the reduction in the joint group after treatment was more evident as compared with the control group (*P* < 0.05). The joint group witnessed better memory function, physical function, behavioral function, emotional function, and communication function than the control group (*P* < 0.05), and the scores of all dimensions of the two groups of patients after treatment were superior to those before treatment (*P* < 0.05). The occurrence of side effects was not statistically different between the two groups of patients (*P* > 0.05). The 1-year survival rate of the control group was 41.67%, the PFS was 6.33 ± 1.02 months, and the OS was 15.51 ± 2.16 months; the 1-year survival rate of the joint group was 43.33%, and the PFS was 8.05 ± 1.07 months, and OS was 16.03 ± 2.44 months; there was no significant difference in the 1-year survival rate between the two groups (*P* > 0.05), the difference in PFS between the groups was significant (*t* = 9.013, *P* < 0.001), and the difference in OS between the groups was not significant (*t* = 1.236, *P*=0.219).

**Conclusion:**

Trastuzumab + cisplatin combined with irinotecan yields a promising result in the treatment of advanced gastric cancer. It can effectively regulate the expression level of tumor markers, delay disease progression, and improve the quality of life of patients. Moreover, irinotecan will not bring more toxic side effects.

## 1. Introduction

The latest statistics in 2019 demonstrate that the incidence and mortality of gastric cancer in China rank 2nd and 3rd among all malignant tumors, respectively, surpassing the world average [1, 2]. Early-stage gastric cancer presents insidious symptoms or is accompanied by nausea, mild stomach pain, and other atypical symptoms. Therefore, more than 69% of patients are in the advanced stage of gastric cancer at the time of diagnosis and cannot be treated with surgery. This type of patient not only has a short survival time but also has their quality of life undermined. At present, platinum-based chemotherapeutics combined with trastuzumab have become one of the standard chemotherapy regimens for advanced gastric cancer. In particular, it exerts a targeted regulation effect on the human epidermal growth factor receptor-2 (HER-2) regulated transduction pathway. However, many elderly patients with advanced gastric cancer are prone to toxic side effects of grade III or higher due to physiological decline and the side effects of chemotherapy drugs, or treatment failure due to drug resistance [3–5]. In recent years, with the continuous development of tumor molecular biology technology, the clinical understanding of gastric cancer pathogenesis and drug resistance mechanisms has become increasingly clear. Irinotecan is a DNA topoisomerase I inhibitor, which can treat tumors by inhibiting the mitosis of tumor cells and is suitable for advanced cancer chemotherapy [6–9]. As previously noted, CEA positivity was associated with lymph node involvement; the decrease of CA72-4 after neoadjuvant chemotherapy could predict pathologic response; elevated CA19-9 levels appeared to be of disease control after neoadjuvant chemotherapy. The measurement of these tumor markers might be useful in the monitoring of response and in the prediction of prognosis in patients treated with neoadjuvant chemotherapy. At present, there is a paucity of reports on trastuzumab + cisplatin combined with irinotecan in the treatment of advanced gastric cancer. Therefore, this study set out to explore trastuzumab + cisplatin combined with irinotecan for disease control of advanced gastric cancer to guide clinical treatment by selecting patients in our hospital who met the research standards.

## 2. Research Method

### 2.1. Case Selection and Grouping

A total of 120 patients with advanced gastric cancer admitted to our hospital from January 2018 to January 2021 were selected and divided into a control group and a combination group according to the treatment plan, with 60 cases in each group; the control group was treated with trastuzumab + cisplatin; on this basis, the joint group was treated with irinotecan; the study was carried out with the approval of the ethics committee of Cangzhou Central Hospital, Approval no. 209001. All subjects gave written informed consent in accordance with the Declaration of Helsinki.

### 2.2. Inclusion Criteria

Inclusion criteria were defined as follows: ①according to the results of gastroscopy, imaging data, pathological examination, molecular biological testing, and serum index examination, all patients met the clinical diagnostic criteria for advanced gastric cancer in the *Chinese Standards for the Diagnosis and Treatment of Common Malignant Tumors* [10]; ② had indications for touzumab + cisplatin therapy; ③ newly treated; ④ expected survival period was not less than 3 months; and ⑤ patients and their family members agreed to participate in this study. In addition, HER-2 positivity was defined as IHC3+ or IHC2+ and in situ hybridisation (ISH)-positive (ISH positivity was defined as a HER-2: CEP17 signal ratio of ≥2.0) by central testing.

### 2.3. Exclusion Criteria

Exclusion criteria were defined as follows: ①combined with severe cardiovascular disease, liver and kidney disease, coagulopathy, mental disorder, infection, and other diseases; ②combined with other gastric diseases or other malignant tumors; ③patients who have undergone surgical treatment; and ④failed to complete follow-up due to certain factors.

## 3. Method

### 3.1. Control Group: Trastuzumab + Cisplatin Treatment

Intravenous infusion of trastuzumab (specification: 440 mg/bottle, manufacturer: Shanghai Roche, approval number S20110007) was given from the first day of treatment, 8 mg/kg, and then on the first day of each treatment cycle, intravenous infusion of trastuzumab was given, 6 mg/kg [11–13]. On days 1 to 3 of treatment, an intravenous infusion of cisplatin (specification: 10 mg/bottle, Qilu Pharmaceutical Co., Ltd., approval number H37021358) was given at 30 mg/m^2^.

### 3.2. Combination Group: Trastuzumab + Cisplatin + Irinotecan Treatment

On the basis of the control group, an intravenous drip of irinotecan hydrochloride injection (specification: 40 mg/bottle, manufacturer: Qilu Pharmaceutical Co., Ltd., National Medicine Standard H20084571) was given at 60 mg/m^2^.

21 days is a treatment cycle, and both groups of patients were treated for 2 consecutive cycles.

### 3.3. Observation Indicators

General information: After the patients were admitted to the hospital, their age, gender, TNM stage, location of onset, tumor type, and other general information were statistically processed.

Clinical efficacy: according to Response Evaluation Criteria in Solid Tumors (RECIST) [4], the clinical efficacy was evaluated. If the patient's lesions disappeared for more than 1 month, it is considered as complete remission (CR); if tumor maximum diameter × maximum vertical diameter decreased by 50% or more, and continued for more than 1 month, it is regarded as partial remission (PR); if tumor maximum diameter × maximum vertical diameter was reduced by less than 50%, or increased by less than 25%, it is deemed as stable disease (SD); if the value of tumor maximum diameter × maximum vertical diameter increased by 25% or above, or the new lesions occurred, it is defined as disease progression (PD); objective effective rate (ORR) = (CR + PR)/total × 100%; and disease control rate (DCR) = (CR + PR + SD)/total × 100%.

Tumor markers: 3 ml of fasting venous blood was collected from the patient, centrifuged at 3000 r/min for 10 min, then the serum was obtained; the carbohydrate antigen 199 (CA199), carcinoembrysis antigen (CEA), and carbohydrate antigen 724 (CA724) of the patient were detected by chemiluminescence using an automatic biochemical analyzer (model: A720).

Quality of life [14]: a quality of life scale was used to assess the quality of life of the two groups of patients before and after treatment, including five dimensions of memory function, physical function, behavior function, emotional function, and communication function, with full score of 100 points for each item; the score is directly proportional to the patient's quality of life; the side effects of chemotherapy drugs were evaluated with reference to the WHO's unified standards.

Long-term efficacy: ① 1-year survival rate = number of survived cases one year after treatment/total number of cases enrolled in treatment × 100%; ② disease progression-free survival (PFS): time interval from the beginning of treatment to the discovery of tumor progression; ③overall survival (OS): the time from treatment to death of the patient. All patients were followed up for at least one year, and the frequency of follow-up was once every two weeks.

### 3.4. Statistical Analysis

In this study, the software SPSS22.0 was used for data analysis and the software GraphPad Prism 7 (GraphPad Software, San Diego, USA) for graphics plotting. The results comprised counting and measurement data, which were expressed in the form of [n (%)] and (*x* ± *s*), respectively, and analyzed by *X*^2^ and *t*-test. A *P* value of <0.05 indicates that there is a statistical difference.

## 4. Results

### 4.1. General Information

There was no difference between the two groups in general data such as age, gender, TNM stage, location of onset, and tumor type (*P* > 0.05). See Table 1.

### 4.2. Clinical Efficacy

After treatment, there were 4 patients with CR in the joint group and 0 patients with CR in the control group. The ORR and DCR of the joint group were significantly higher than those of the control group (*P* < 0.05), as shown in Table 2.

### 4.3. Tumor Marker Levels

**Figure 1:**
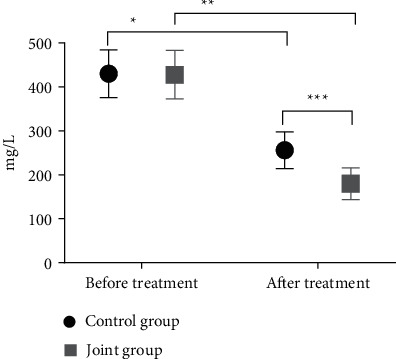
Comparison of the CA199 levels of the two groups of patients. Note: The abscissa represents before and after treatment, and the ordinate represents the expression level, mg/L; the expression levels of CA199 in the control group before and after treatment were 430.15 ± 54.18 and 255.94 ± 41.73; the expression levels of CA199 before and after treatment in the combination group were 427.88 ± 55.12 and 179.80 ± 36.15; ^*∗*^indicating that the expression level of CA199 in the control group was significantly different before and after treatment (*t* = 19.732, *P* < 0.001); ^*∗∗*^indicates that the expression level of CA199 in the joint group before and after treatment was significantly different (*t* = 29.152, *P* < 0.001); ^*∗*∗∗^indicates that the CA199 expression level after treatment between the two groups was significantly different (*t* = 10.682, *P* < 0.001).

**Figure 2:**
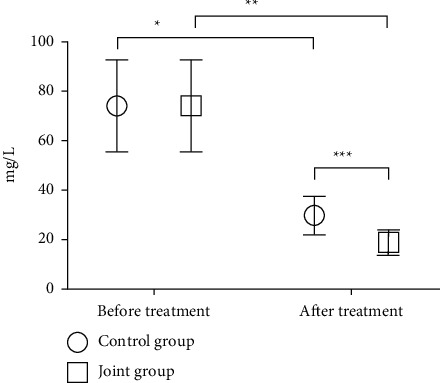
Comparison of the CEA levels of the two groups of patients. Note: The abscissa represents before and after treatment, and the ordinate represents the expression level, mg/L; the expression levels of CEA before and after treatment in the control group were 74.23 ± 18.65 and (29.76 ± 7.85); the expression levels of CEA before and after treatment in the combination group were 74.05 ± 18.44 and 19.07 ± 5.09; ^*∗*^indicates that the CEA expression levels of the control group patients before and after treatment were significantly different (*t* = 17.023, *P* < 0.001); ^*∗∗*^indicates that the CEA expression levels of patients in the combination group before and after treatment were significantly different (*t* = 22.263, *P* < 0.001); ^*∗*∗∗^indicates that the CEA expression levels of the two groups of patients after treatment were significantly different (*t* = 8.851, *P* < 0.001).

**Figure 3:**
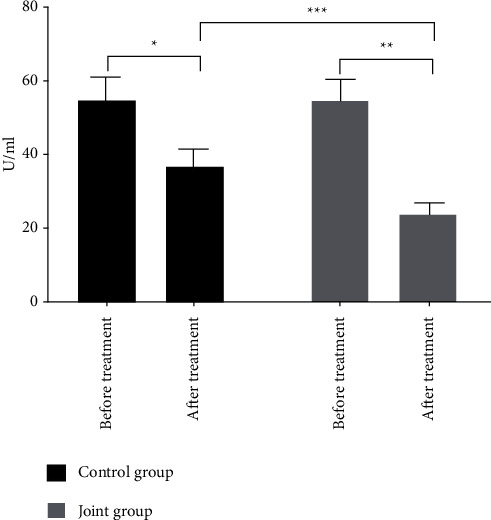
Comparison of the CA724 levels of the two groups of patients. Note: The abscissa indicates before and after treatment, and the ordinate indicates the expression level, U/ml; the expression levels of CA724 in the control group before and after treatment were 54.78 ± 6.16 and 36.84 ± 4.71; the expression levels of CA724 in the joint group before and after treatment were 54.53 ± 6.08 and 23.88 ± 3.15; ^*∗*^indicates that the expression level of CA724 in the control group was significantly different before and after treatment (*t* = 17.921, *P* < 0.001); ^*∗∗*^indicates that the expression level of CA724 in the joint group was significantly different before and after treatment (*t* = 34.671, *P* < 0.001); ^*∗*∗∗^indicates that the expression level of CA724 after treatment between the two groups was significantly different (*t* = 17.717, *P* < 0.001).

The expression levels of CA199, CEA, and CA724 after treatment in the two groups were significantly reduced (*P* < 0.05), and the reduction in the joint group after treatment was more evident as compared with the control group (*P* < 0.05) (see Figures 1–3).

### 4.4. Quality of Life

The joint group witnessed better memory function, physical function, behavioral function, emotional function, and communication function than the control group (*P* < 0.05), and the scores of all dimensions of the two groups of patients after treatment were superior to those before treatment (*P* < 0.05). See Table 3.

### 4.5. Toxic and Side Effects

The occurrence of side effects was not statistically different between the two groups of patients (*P* > 0.05, Table 4).

### 4.6. Long-Term Efficacy

**Figure 4:**
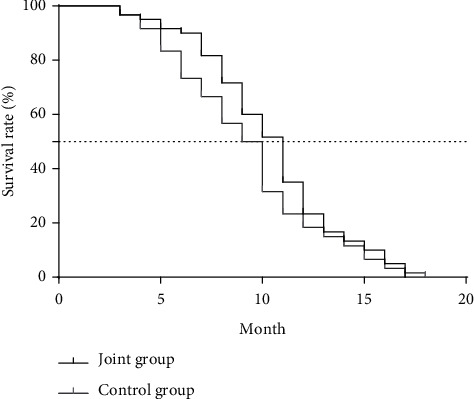
Comparison of PFS between the two groups of patients.

**Figure 5:**
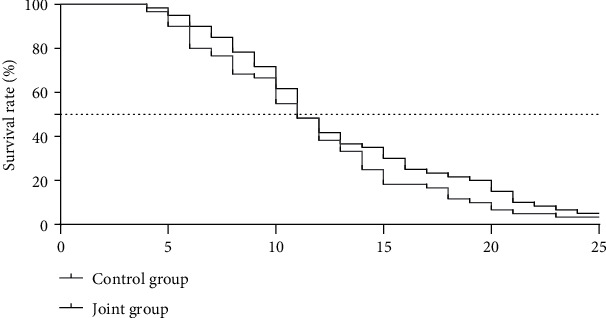
Comparison of OS between the two groups of patients.

The 1-year survival rate of the control group was 41.67%, the PFS was 6.33 ± 1.02 months, and the OS was 15.51 ± 2.16 months; the 1-year survival rate of the joint group was 43.33%, the PFS was 8.05 ± 1.07 months, and the OS was 16.03 ± 2.44 months; there was no significant difference in the 1-year survival rate between the two groups (*P* > 0.05), the difference in PFS between the groups was significant (*t* = 9.013, *P* < 0.001), and the difference in OS between the groups was not significant (*t* = 1.236, *P*=0.219), as presented in Table 5, Figures 4 and 5.

## 5. Discussion

Gastric cancer is a common malignant tumor in clinical practice. It is estimated that up to 550,000 deaths are consequent of gastric cancer in China every year. In recent years, the gastric cancer death rate presents an increasing trend year by year, seriously threatening human life and health [15]. Generally, early gastric cancer is relatively insidious, and most patients are already at an advanced stage when they are diagnosed and thus cannot be removed by surgery [16–18]. Therefore, how to improve the disease control rate of patients with advanced gastric cancer is the focus of clinical research at this stage. HER-2 is a member of the epidermal growth factor receptor family and is highly expressed in the bodies of gastric cancer patients. This substance can regulate the peritoneal metastasis and lymph node metastasis of cancer cells and then aggravate the proliferation, infiltration, and metastasis of malignant tumor cells. Therefore, our hospital selects anti-HER-2 drugs stipulated by the updated 2021 CSCO guidelines for the diagnosis and treatment of metastatic gastric cancer, namely, trastuzumab combined with cisplatin, to treat advanced gastric cancer. In order to further improve the disease control rate of patients with advanced gastric cancer, our hospital has added Irinotecan with the purpose of improving the clinical efficacy of advanced gastric cancer.

Irinotecan is a semisynthetic water-soluble camptothecin derivative, which can specifically inhibit DNA topoisomerase I and can be metabolized by carboxylesterase to SN-38 in most tissues, and SN-38 is stronger than that of irinotecan, and both SN-38 and irinotecan can induce single-stranded DNA damage and block DNA replication [19, 20]. In addition, irinotecan has shown broad-spectrum antitumor activity against mouse tumor models and has antihuman xenograft tumor activity. Also, it has antitumor activity against tumors expressing P-glycoprotein MDR. However, the insensitivity of chemotherapeutic drugs and their adverse reactions to the body have always been a problem that plagues clinical treatment. But so far, the clinical application of irinotecan has become more and more mature, and its adverse reactions are predictable and controllable. At the same time, the addition of irinotecan to the conventional chemotherapy regimen in this study can better increase the sensitivity of the treatment of advanced gastric cancer.

Similar to studies by SASAKI [21] and SATAKE [22], the present study concluded that the ORR and DCR of the joint group were significantly higher than those of the control group; the expression levels of CA199, CEA, and CA724 were significantly reduced; and the expression levels of CA199, CEA, and CA724 in the joint group were significantly lower than those in the control group. Fortunately, there was no significant difference in the occurrence of side effects between the two groups of patients. In terms of short-term efficacy, trastuzumab + cisplatin can effectively control the lesions in patients with gastric cancer and effectively reduce the expression levels of tumor markers such as CA199, CEA, and CA724, but the combination of the two combined with irinotecan yields a more significant effect on advanced gastric cancer, and it is safe without obvious side effects.

In terms of long-term efficacy, the quality of life scores of memory function, physical function, behavioral function, emotional function, and communication function of the joint group were significantly better than those of the control group, and the scores of each dimension of the two groups of patients after treatment were better than those before treatment; the 1-year survival rate of the control group was 41.67%, the PFS was 6.33 ± 1.02 months, and the OS was 15.51 ± 2.16 months; the 1-year survival rate of the joint group was 43.33%, the PFS was 8.05 ± 1.07 months, and the OS was 16.03 ± 2.44 months. There was no statistical difference in the 1-year survival rate between the two groups. This result shows that the combined application of irinotecan on the basis of trastuzumab + cisplatin can effectively delay the progression of the disease and significantly improve the control rate of the lesion, thereby winning a valuable time window for the subsequent treatment plans.

In summary, trastuzumab + cisplatin combined with irinotecan emanates a prominent result in the treatment of advanced gastric cancer. It can effectively regulate the expression level of tumor markers, delay disease progression, and help improve the quality of life of patients. Despite the fact that this study has made an innovation in the adjustment of treatment plan, the sample size of the study is small, and it is a single-center study, which may also be one of the reasons for the insignificant difference in OS. On account of these, the sample still needs to be expanded in the future, and in-depth research should be carried out to create more possibilities for improving the survival rate and overall survival time to guide clinical treatment.

## Figures and Tables

**Table 1:** Comparison of general information of the two groups of patients (*n* = 60).

	Control group	Joint group	*X* ^2^/*t*	*P*
Age (years)	59.86 ± 4.72	60.14 ± 5.03	0.314	0.754
Male/female	41/19	38/22	0.333	0.564
*TNM staging*				

IIIa	15 (25)	14 (23.33)	0.046	0.831
IIIb	21 (35)	20 (33.33)	0.037	0.847
IV	24 (40)	26 (43.33)	0.137	0.711
*Disease site*				

Fundus of stomach	26 (43.33)	25 (41.67)	0.034	0.853
Gastric antrum	23 (38.33)	25 (41.67)	0.139	0.709
Cardia	11 (18.33)	10 (16.67)	0.058	0.810
*Tumor typing*				

Poorly differentiated diffuse carcinoma	27 (45)	25 (41.67)	0.136	0.713
Moderately differentiated adenocarcinoma	15 (25)	16 (26.67)	0.044	0.835
Poorly differentiated adenocarcinoma	13 (21.67)	12 (20)	0.051	0.822
Hepatoid adenocarcinoma	3 (5)	4 (6.67)	0.152	0.697
Mucinous adenocarcinoma	2 (3.33)	3 (5)	0.209	0.648

**Table 2:** Comparison of the clinical efficacy of the two groups of patients (%).

Groups	*n*	CR	PR	SD	*P*D	ORR	DCR
Control group	60	0	26	24	10	26	50
Joint group	60	4	33	20	3	37	57
*X* ^2^						4.043	4.227
*P*						0.044	0.040

**Table 3:** Comparison of the quality-of-life scores of the two groups of patients.

Dimensions	Memory function		Body function		Behavioral function		Emotional function		Communicative function	
	Before treatment	After treatment	Before treatment	After treatment	Before treatment	After treatment	Before treatment	After treatment	Before treatment	After treatment
Control group	15.16 ± 2.35	56.11 ± 5.62	16.25 ± 2.51	58.17 ± 6.05	13.97 ± 2.44	44.72 ± 5.30	17.26 ± 2.58	55.26 ± 6.15	14.63 ± 2.27	50.23 ± 5.19
Joint group	14.86 ± 2.41	77.81 ± 8.03	16.18 ± 2.46	70.56 ± 7.71	14.06 ± 2.83	70.12 ± 6.85	17.33 ± 3.01	72.59 ± 8.01	15.02 ± 2.35	69.58 ± 7.13
*t*	12.010	17.150	11.202	9.793	10.031	22.717	9.020	13.293	7.521	16.996
*P*	0.372	<0.001	0.802	<0.001	0.600	<0.001	0.701	<0.001	0.865	<0.001

**Table 4:** Comparison of the occurrence of side effects of the two groups of patients.

	Control group (*n* = 60)				Joint group (*n* = 60)			
	I	II	III	Total incidence (%)	I	II	III	Total incidence (%)
Liver toxicity	1	2	1	4 (6.67)	2	1	1	4 (6.67)^*∗*^
Kidney toxicity	2	1	0	3 (5)	1	2	0	3 (5)^*∗*^
Neurotoxicity	1	2	0	3 (5)	1	0	1	2 (3.33)^*∗*^
Cardiotoxicity	1	3	0	4 (6.67)	1	2	0	3 (5)^*∗*^
Bone marrow suppression	3	1	1	5 (8.33)	2	1	1	4 (6.67)^*∗*^
Gastrointestinal reaction	6	3	2	11 (18.33)	5	4	3	12 (20)^*∗*^
Allergic reaction	2	0	0	2 (3.33)	1	0	1	2 (3.33)^*∗*^

^*∗*^Indicates that the difference is not significant compared with the total incidence of the control group (*P* > 0.05).

**Table 5:** Comparison of the 1-year survival rate of the two groups of patients.

Groups	*n*	Number of survived	Survival rate (%)
Control group	60	25	41.67
Joint group	60	26	43.33
*X* ^2^		0.034	
*P*		0.853	

## Data Availability

All data generated or analyzed during this study are included in this published article.
